# Port implantation-related bloodstream infection caused by *Wickerhamomyces myanmarensis*: A case report

**DOI:** 10.18502/CMM.2023.150671

**Published:** 2023-03

**Authors:** Ali Aminasnafi, Sadegh Khodavaisy, Maryam Moslem, Marziye Esmaeilpour Jouneghani, Fatemeh Sarbandi, Mahsa Falahatinejad, Bahareh Bashardoust, Hamid Badali, Neda Kiasat

**Affiliations:** 1 Health Research Institute, Research Center of Thalassemia & Hemoglobinopathy, Ahvaz Jundishapur University of Medical Sciences, Ahvaz, Iran; 2 Department of Medical Parasitology and Mycology, School of Public Health, Tehran University of Medical Sciences, Tehran, Iran; 3 Research Center for Antibiotic Stewardship and Antimicrobial Resistance, Tehran University of Medical Sciences, Tehran, Iran; 4 Department of Medical Mycology, School of Medicine, Ahvaz Jundishapur University of Medical Sciences, Ahvaz, Iran; 5 Imam Khomeini Hospital, Ahvaz Jundishapur University of Medical Sciences, Ahvaz, Iran; 6 Department of Pediatrics, School of Medicine, Ahvaz Jundishapur University of Medical Sciences, Ahvaz, Iran; 7 Department of Medical Mycology, Tarbiat Modares University, Tehran, Iran; 8 Department of Molecular Microbiology and Immunology, South Texas Center for Emerging Infectious Diseases, The University of Texas, San Antonio, Texas, USA

**Keywords:** Bloodstream infections, *Wickerhamomyces myanmarensis*, Voriconazole

## Abstract

**Background and Purpose:**

*Wickerhamomyces myanmarensis* is a new opportunistic yeast previously named *Pichai myanmarensis*, which belongs to the order Saccharomycetales. Since its discovery, one environmental isolate of *W. myanmarensis* has been reported from Myanmar, and one clinical sample from Iran.

**Case Report:**

We report a case of bloodstream infection related to an implantable venous access port. *W. myanmarensis* was isolated from patient's blood after chemotherapy, which was meant to control and heal T-cell lymphoblastic lymphoma. Broth dilution minimum inhibitory concentrations were performed according to the CLSI M27-A3 document. The patient recovered with intravenous voriconazole and was discharged with the recommended prescription of oral voriconazole as a maintenance drug.

**Conclusion:**

So far, only one case of *W. myanmarensis* fungemia has been reported in the world in 2019. This is the second case of bloodstream infection with this yeast from a patient undergoing chemotherapy in Iran.

## Introduction

Venous port implantation is meant for cancer patients to inject chemotherapy drugs. Various studies have reported that implantable venous access port (IVAP) can cause major side effects related to bloodstream infections (BSIs) with a rate ranging from 2.4-16.4% [ 1 - 4 ]. *Wickerhamomyces myanmarensis* is a novel opportunistic human pathogen. This yeast, formerly known as *Pichia myanmarensis*, was first isolated in 2000 by Nagatsuka et al. from palm sugar on a warehouse floor at Rum Distillery in Mandalay, Myanmar. Now, this yeast has been grouped in the genus *Wickerhamomyces*, family *Wickerhamomycetaceae*, and order *Saccharomycetales*. White to cream, smooth, and glossy colonies of *W. myanmarensis* grow at 37℃ and 40℃ [ 5 , 6 ]. This specie is a multipolar budding yeast cell that does not produce pseudohyphae on cornmeal agar. *W. myanmarensis* is a urease-negative yeast that contains ubiquinone Q-7 and does not grow in the presence of 0.01 or 0.1% cycloheximide. Literature on the clinical significance of *W. myanmarensis* is scarce. However, this yeast appears to be an opportunistic agent with high similarity to *W. anomalus*, which highlights the importance of detecting *W. myanmarensis* because of numerous reports of clinical cases and mortality caused by *W. anomalus* [ 7 ]. This paper will present a case of *W.myanmarensis* causing IVAP-related BSI in a hospitalized child with T-cell lymphoblastic lymphoma. 

## Case Report

**Figure 1:**
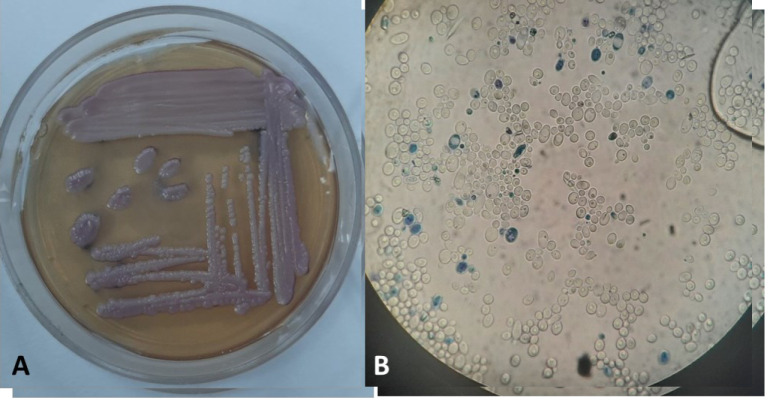
culture and direct examination of *Wickerhamomyces myanmarensis*. (A) *W. myanmarensis* growth on CHOROMagar *Candida* medium (72h), (B) Budding yeast cells in wet mount examination at 1000x magnification

A 4-year-old girl with T-cell lymphoblastic lymphoma was admitted for chemotherapy at Shahid Baghaei Hospital, Ahvaz, Iran. On the first day of admission (day 0), the patient had no symptoms but as soon as a needle entered IVAP to inject normal saline, her body temperature rose to 39°C (102°F). Due to the presence of fever, antibiotic therapy started with cefepime (50 mg per kg) every 8 hours to diminish the febrile status. During this period, the erythrocyte sedimentation rate (ESR) of the patient was equal to 47, and C-reactive protein (CRP) was three plus (+3) with WBC count of 2500/µl. After 48 hours of antibiotic therapy, the fever did not improve. On the third day of hospitalization, a blood culture from the port was sent to the medical mycology laboratory for fungal detection. White to cream, smooth, and glossy yeast colonies grew on Sabouraud dextrose agar medium (Merck Co., Darmstadt, Germany) at 37℃ but no hyphae or pseudohyphae was observed in wet mount examination (Figure 1A and B).

Genomic DNA of isolate was extracted using SinaPure^TM^DNA kit (SinaClonBioScience, Tehran, Iran) according to the manufacturer’s instructions. The internal transcribed spacer 1 (ITS1) and ITS2 regions and the 5.8S ribosomal DNA (rDNA) region of the fungi were amplified and sequenced by using universal primers ITS1/ITS4. Using BLAST analysis, the ITS sequences of isolate exhibited 100% homology with *W. myanmarensis*. Sequence with 778bp length was deposited in GenBank with the accession no. OQ455736. The patient was treated with intravenous voriconazole at a dosage of 9 mg on the first day and then 6 mg on the second day. After taking voriconazole for 48 hours, the fever was resolved in the patient. However, the treatment continued with intravenous voriconazole for seven days, namely the time the antifungal susceptibility profile became available. *In vitro* antifungal susceptibility testing (AFST) was performed using the broth microdilution CLSI M27-A3 method [ 8 ]. Antifungals (Sigma-Aldrich, St. Louis, MO, USA) used in this study had MICs for amphotericin B, 0.25 μg/ml; anidulafungin, ≤0.015 μg/ml; micafungin, 0.06 μg/ml; caspofungin, 0.125 μg/ml; fluconazole, 2 μg/ml; voriconazole, ≤0.03 μg/ml; posaconazole, 0.25 μg/ml; isavuconazole,0.06 μg/ml; ibrexafungerp, 0.25 μg/ml; Itraconazole,0.125 μg/ml (Table 1). There are no interpretative breakpoints available for *W. myanmarensis*. On the 7^th^ day of hospitalization and during maintenance of therapy, two blood cultures from the vein and port were sent to the medical mycology laboratory for monitoring of the treatment regimen, and the results of both were negative. Fortunately, after seven days of treatment, there was a significant improvement in laboratory indices such as ESR (8) and CRP (negative) with 9.7/ µL WBC count without fever. Eventually, after normal results of echocardiography, brain Magnetic Resonance Imaging (MRI), abdominal and pelvic ultrasound scanner, the patient was discharged with voriconazole maintenance therapy. On the first day, treatment started with intravenous voriconazole at a dosage of 9 mg/kgevery 12 h, then 6 mg/kg every 12 h for one week, followed by oral voriconazole at a dosage of 6 mg/kg every 12 h for two weeks.

**Table 1:** Antifungal susceptibility testing of *Wickerhamomyces myanmarensis* isolated from different sources in Iran and/or global distribution

Isolation sourc	FLC (µg/ml)	I TC(µg/ml)	VRC (µg/ml)	PSC (µg/ml)	ISA (µg/ml)	IBX (µg/ml)	AMB (µg/ml)	MCF (µg/ml)	CAS (µg/ml)	ANF (µg/ml)
Our case	2	0.125	≤0.03	0.25	0.06	0.25	0.25	0.06	0.125	≤0.015
Blood/Shiraz	16	0.06	1	0.5	-	-	2	-	-	-
Central venous catheter/Shiraz	0.5	0.06	0.06	0.125	-	-	2	-	-	-

Abbreviations: FLC, Fluconazole; ITC, Itraconazole; VRC, Voriconazole; PSC, Posaconazole; ISA, Isavuconazole; IBX, Ibrexafungerp; AMB, Amphotericin B; MCF, Micafungin; CAS, Caspofungin; ANF, Anidulafungin

## Discussion

Candidemia in children afflicted with malignancy is usually associated with high mortality rates [ 9 , 10 ]. Until today, the majority of candidemia cases are caused by common Candida species (albicans and non-albicans), and the contribution of rare species to this disease is weak. However, early and accurate diagnosis of rare species is of particular importance for various reasons, including resistance to antifungals, advances in medical care, increasing number of immunocompromised patients, and development of new treatments [ 11 , 12 ]. *W. myanmarensis* is a rare non-albicans environmental yeast species that was first reported from palm sugar in Myanmar (2005) [ 6 ]. So far, the only clinical case of this yeast has been isolated from the blood and central venous catheter of a 5.5-year-old boy in Shiraz, Iran (2017) [ 7 ]. In this article, we report the second clinical case of *W. myanmarensis* in a 4-year-old girl with an underlying disease (lymphoma). The patient was admitted for chemotherapy with general symptoms of weakness related to the disease but without symptoms of septicemia. Considering the COVID-19 pandemic, the patient was discharged from the hospital after completing each course of treatment, so long hospital stays, which is one of the most important factors of fungemia, was not true in this case. Since the isolates were recovered only from IVAP, the blood cultures were negative on three different days. Hence, the exogenous source of infection was suspected, namely candidemia associated with the contaminated port because *W. myanmarensis* is an environmental yeast, and only few isolates of it have been recovered from a clinical setting [ 13 ]. On the other hand, the symptoms associated with candidemia were not resolved by replacing the port, whereas the blood culture still was negative after replacement. The result of blood culture was likely to be false negative due to the low sensitivity of traditional blood culture method (not BACTEC system). Unfortunately, we could not take samples from the hands of healthcare workers, medical equipment, and the environment. Realizing that the first isolation of this yeast was from dates and banana juice and because of the presence of *W. anomalous*, a closely related species used in the biotechnology and winemaking industries [ 13 ], we investigated the patient's nutrition, but none of the possible sources before and after candidemia was not included in patient's diet. In the only similar study to ours, Arastefar et al. mentioned the use of honey and the involvement of intestinal mucosa [ 7 ]. We cannot claim that the contaminated source is port, although this possibility is stronger for us, while we cannot support the diet source of yeast. Optimal management of invasive fungal infections depends on correct antifungal therapy. Prophylaxis and inappropriate treatments in patients with underlying diseases are the reasons for the emergence of rare fungal species. So far, there are no guidelines for performing and interpreting AFST for *W. myanmarensis*. We did not find strong documentation indicating low virulence of *W. myanmarensis*. Hence, given the underlying disease of lymphoma, immunodeficiency, and frequent chemotherapies, antifungal treatment was prescribed after the port was removed. Our AFST results for *W. myanmarensis* showed the lowest MIC value for anidulafungin (≤0.015), followed by voriconazole (≤0.03). According to several studies, echinocandins are recommended as the first line of treatment for invasive candidiasis [ 14 , 15 ]. Due to the absence of anidulafungin in Iran as well as the high cost of therapy, treatment with voriconazole was considered an alternative to echinocandin that led to a good outcome in our patient. Surprisingly, we observed that voriconazole susceptibility was favorable in *W. myanmarensis*, which was consistent with the study of Arastefar et al. in the yeast isolated from the central venous catheter, although they reported resistance to voriconazole for the same yeast isolated from blood [ 7 ]. This may be due to differences in clones, the immune system's response to yeast in the blood, the port, or disease severity. Perhaps *W. myanmarensis* is a novel opportunist yeast species more common than reported cases that has remained unidentified up to now. The study of features *W. myanmarensis* such as virulence and risk factors as well as other microscopic and macroscopic characteristics of this organism would be crucial for accurate diagnosis by laboratory personnel and effective treatment of a patient infected with *W. myanmarensis* by clinicians.

## Conclusion

The presence of *W. myanmarensis* in bloodstream infection is considered an emerging nosocomial threat. According to the report of this case as the second *W. myanmarensis* identified from Iran, more research about this yeast is needed to establish proper management guidelines in suspicious cases. On the other hand, due to the environmental niche of *W. myanmarensis*, strict adherence to hygiene and disinfection protocols is of great importance to prevent the colonization of this opportunistic yeast in hospital wards.

## Acknowledgments

We would like to thank the pediatrics department of Baghaei 2 Hospital and Ahvaz Jundishapur University of Medical Sciences for their support.

## Authors’ contribution

Design and technical supervision: NK, AAA, and S.K. Sampling and interpretation: ME and FS. Technical support: HB. Data collection and manuscript preparation: MM and NK. Critical review of the manuscript: BB and MF. All authors have read and approved the submitted final manuscript.

## Conflicts of interest

The authors declare that they have no potential conflicts of interest.

## Financial disclosure

This study was funded by a grant (No. TH-0105) from Ahvaz Jundishapur University of Medical Sciences, Ahvaz, Iran.

## Ethics statement

This project was approved by the ethical committee of Ahvaz Jundishapur University of Medical Sciences (Registered Code IR.AJUMS. HGOLESTAN.REC.1401.029).
